# Biometric risk factors for myopia onset in emmetropic school-age children

**DOI:** 10.1007/s10384-025-01222-2

**Published:** 2025-06-03

**Authors:** Yoshinori Nakai, Osamu Hieda, Yo Nakamura, Mitsuko Nakata, Chie Sotozono, Shigeru Kinoshita

**Affiliations:** 1 Department of Ophthalmology, Kyoto Prefectural University of Medicine, 465 Kajii-cho, Hirokoji-agaru, Kawaramachi-dori, Kamigyo-ku, Kyoto, 602-0841 Japan; 2 Department of Biostatistics, Kyoto Prefectural University of Medicine, Kyoto, Japan; 3 Department of Frontier Medical Science and Technology for Ophthalmology, Kyoto Prefectural University of Medicine, Kyoto, Japan

**Keywords:** School children, Myopia onset, Higher-order aberrations, Axial length, Sex

## Abstract

**Purpose:**

To identify the potential biometric risk factors for the onset of myopia in emmetropic school-age children.

**Study design:**

Longitudinal study.

**Methods:**

First, we performed a preliminary study in which objective refraction, corneal refractive power, higher-order aberrations (HOAs), and axial length (AL) was measured annually in 98 Grade-3 (age 8) elementary schoolchildren over a 4-year period from 2006 to 2009. We also examined the refractive changes over 3 years, and assessed the correlation between those changes and the baseline data. Based on those findings, we performed the primary study in which objective and subjective refraction, corneal refractive power, HOAs, and AL was measured annually in Grade 1 (age 6) through Grade 8 (age 13) schoolchildren from 2013 to 2022. We investigated the risk factors for AL elongation over 1 year in children with emmetropia at the first year using a multivariable linear mixed model.

**Results:**

Findings in the preliminary study revealed that AL in the first year (age 8) had the strongest correlation with myopia progression for 3 years. The risk factors for AL elongation among emmetropia were 1) lower grade, 2) sex (female), 3) myopic objective refraction (spherical equivalent), 4) longer AL (mm), 5) lower corneal coma-like aberration (CA) at the pupil diameter of 6 mm, and 6) higher ocular spherical aberration (SA) of 4 mm and lower ocular SA at the pupil diameter of 6 mm in the primary study.

**Conclusion:**

Risk factors for myopia onset in emmetropic school-age children include AL, refraction, corneal CAs, and ocular SAs.

**Supplementary Information:**

The online version contains supplementary material available at 10.1007/s10384-025-01222-2.

## Introduction

According to the findings in the Japanese School Health Examination Survey (http://www.e-stat.go.jp/) conducted by the Japanese Ministry of Health, Labour and Welfare, the prevalence of myopia is increasing in elementary-grade school children in Japan. The percentage of these children with a visual acuity (VA) of less than decimal 1.0 reached 37.9% in 2022. Although it has been reported that myopia is more common in Asia than in other regions of the world [1, 2], the findings in recent studies show that its prevalence is currently increasing in both Europe and the United States [3–6] as well. Moreover, the findings in several large studies indicate that the risk factors for myopia onset and progression are genetic predisposition and environmental factors such as outdoor activities and near-vision work [7–11]. High myopia is a risk factor for the development of chorioretinal atrophy, macular neovascularization, and glaucoma, all of which can lead to potentially blinding ocular conditions [12]. The current increase in the myopic population may result in a greater number of patients with high myopia, so it is vital that preventive measures be taken against the onset and progression of myopia [13].

Treatment options proposed for the prevention of myopia progression include orthokeratology, low-concentration atropine eye drops, and the use of multifocal contact lenses [6, 14–18]. It is, therefore, becoming more important, to detect risk factors of myopia onset among emmetropic children. Currently, factors known to inhibit the progression of myopia are low-concentration atropine eye drops, red light therapy, and outdoor activity [19–23].

The purpose of this multi-level study (a preliminary study followed by a primary study) was to identify the potential biometric risk factors for myopia onset in emmetropic school-age children.

## Subjects and methods

### (1) Preliminary study

The preliminary study included children attending the Dodo Elementary School in the 'Yamashina-ward of Kyoto who underwent four consecutive annual measurements from Grade 3 (age 8) to Grade 6 (age 11) from 2006 to 2009. The criteria for inclusion in this study were children able to undergo 4 consecutive annual measurements (1 per year). A total of 99 school children, who were not transferred to other schools, were measured annually, 1 child was excluded from the study due to unreliable test results (the exclusion criteria).

In the preliminary study, we measured objective spherical equivalent (SE), corneal refractive power, ocular higher-order aberration (HOA) in a 4 mm pupil diameter, and axial length (AL) in Grade-3 elementary school children annually over a 4-year period from 2006. In this study, we set up all ophthalmological instruments in the school classroom. Examinations were performed by skilled staff and attending pediatric ophthalmologists. Objective SE and HOAs were measured simultaneously using the KR-9000PW^®^ Autorefractor Keratometer/Wavefront Analyzer (Topcon Corporation). AL measurements were obtained by the IOLMaster^®^ (Carl Zeiss Meditec AG).

### (2) Primary study

After the preliminary study, the primary study (Kyoto childhood Refractive Error Study [KRES]) was conducted to examine refractive errors in school children of elementary and junior high schools in Kyoto (Ryofu-Gakuen in the Minami-ward of Kyoto City and Yakuno Elementary School in Fukuchiyama City) from 2013 to 2022.

In the primary study, we analyzed the data of the KRES obtained annually from Grade 1 (age 6) to Grade 9 (age 14). We selected cases with a follow-up period of 2 years and with emmetropia in the first year. We defined emmetropic children as subjective refraction of more than -0.5D and less than 0.5D. Cases with a follow-up period of over 2 years were included again if they remained emmetropic in subsequent years. Therefore, the same cases had been selected numerous times. The inclusion criteria were children who underwent 2 consecutive annual measurements, while the exclusion criterion was a too large amount of HOAs; over 1.0 μm in a 4-mm-diameter pupil and over 2.0 μm in a 6-mm-diameter pupil.

In the primary study, as in the preliminary study, we used various apparatuses and conducted measurements annually in August (Kyoto) and October (Fukuchiyama). We measured objective and subjective refraction, uncorrected VA (UCVA), best-corrected VA (BCVA), AL, and corneal and ocular HOAs in 4-mm and 6-mm pupil diameter measurements. The WR-5100K Binocular Auto Ref/Keratometer^®^ (Grand Seiko) was used for measuring objective refraction. The measurements were taken one eye at a time while looking at a target located 5 meters away. Subjective refraction, UCVA and BCVA were measured at a distance of 5 meters. AL was measured using the IOLMaster^®^ and the IOLMaster^®^ 500 (Carl Zeiss Meditec). HOAs were measured with KR-1W Wavefront Analyzer^®^ (Topcon Corporation). All examinations were performed by orthoptists and ophthalmologists.

### Statistical analysis

In the preliminary study, the changes in AL, objective SE, average K value, and HOAs from the previous grade were tested by paired *t*-test. The correlation between AL and objective SE changes in 3 years were analyzed using the Pearson product-moment correlation coefficient. Moreover, we tested the correlation between AL, objective SE, average K value, and HOAs in Grade 3 and objective SE changes at 3 years using the Pearson product-moment correlation coefficient. Since this was the preliminary study, only the right-eye data was used considering the intra-individual correlation.

In the primary study, to estimate changes in AL over one year while accounting for the correlation between repeated measurements, a linear mixed model was used. The model consisted of the student as a random effect and grade, sex, objective SE, AL, average K value, and HOAs (corneal coma-like, spherical-like, and spherical HOAs at a 4-mm and 6-mm pupil diameter, and ocular coma-like, spherical-like, and spherical HOAs at a 4 mm and 6 mm pupil diameter) in the first year as fixed effects. To efficiently use the full data obtained from both eyes, intra-individual correlation was considered by use of the Huber Sandwich Estimator [24]. To compare effect sizes between the variables, all continuous variables were standardized to have a mean of zero and a standard deviation of one. Data with missing values was excluded (complete case analyses).

### Ethics

The protocols in this multi-level study were approved by the Ethics Committee of Kyoto Prefectural University of Medicine, Kyoto, Japan (Approval No. RBMR-E-467-5). We provided a document detailing the study to the students and their parents, and the parents confirmed that the child's participation was voluntary.

## Results

### (1) Preliminary Study

#### Refraction, AL, corneal refractive power, and HOAs

Annually-measured data from 98 students (53 girls and 45 boys) revealed significant refraction changes and AL over the 3-year period, indicating that myopia progressed from Grade 3. There was no significant increase in corneal refractive power and ocular higher order aberrations (Table 1 and Supplementary Fig. 1). Refraction changes toward myopia and AL elongation over the 3-year period were well correlated (R^2^ = 0.612) (Supplementary Fig. 2).

**Table 1:** Mean findings in the eyes of the 98 Grade 3 (8-years old) through Grade 6 (11-years old) elementary-school children in the preliminary study

Mean Data	Grade 3	Grade 4	Grade 5	Grade 6
AL, mm (SD)	23.07 (±0.85)	23.28 (±0.89)*	23.45 (±0.97)*	23.70 (±1.04)*
Objective SE, D (SD)	−0.53 (±1.1)	−0.71 (±1.05) *	−0.91 (±1.28)*	−1.31 (±1.62) **
Average K, D (SD)	42.62 (±1.63)	43.65 (±1.32)	43.57 (± 134)	43.55 (±1.33)
Coma-like (Z3) O4, μm (SD)	0.068 (±0.03)	0.073 (±0.03)	0.077 (±0.04)	0.083 (±0.04)
Spherical-like (Z4) O4, μm (SD)	0.043 (±0.02)	0.044 (±0.02)	0.042 (±0.02)	0.047 (±0.02)

Findings of the paired* t*-test in comparison to the previous fiscal year; * *P* < 0.05, ** *P* < 0.01

*AL* axial length, *Average K* Average corneal refractive power, *D* diopters, *SE* spherical equivalent, *Z* Zernike order, *O4* 4-mm pupil diameter ocular higher-order aberrations (HOAs), *SD* standard deviation

#### Correlation with myopia progression (objective refraction and AL)

Longer AL at Year 1 (Grade 3) was associated with further AL elongation (*P* < 0.01) and greater refraction changes toward myopia (*P* < 0.01) over the subsequent 3 years (Table 2).

**Table 2:** Correlation between refractive data in Grade 3 and spherical equivalent change

	Correlation coefficient	*P* value
AL, mm	−0.583	< 0.01
SE, D	0.464	< 0.01
Average K, D	0.031	0.815
Coma-like (Z3) O4, μm	−0.053	0.688
Spherical-like (Z4) O4, μm	−0.192	0.821

*AL* axial length, *Average K* Average corneal refractive power, *D* diopters, *SE* spherical equivalent, *Z* Zernike order, *O4* 4-mm pupil diameter ocular higher-order aberrations (HOAs)

### (2) Primary study

#### Risk factors for AL elongation among emmetropic school children

From 882 students (461 boys and 421 girls), a total of 5,384 eyes (2,871 male eyes and 2,513 female eyes) were included in the analysis. The first-year data is shown in Table 3.

**Table 3:** First-year findings in the primary study

Data		Average (standard deviation)*
Sex	Male	2,871 (53.3 %)
	Female	2,513 (46.7 %)
Grade	1 (6 y.o.)	1,154 (21.4 %)
	2 (7 y.o.)	1,092 (20.3 %)
	3 (8 y.o.)	955 (17.7 %)
	4 (9 y.o.)	755 (14.0 %)
	5 (10 y.o.)	590 (11.0 %)
	6 (11 y.o.)	391 (7.3 %)
	7 (12 y.o.)	249 (4.6 %)
	8 (13 y.o.)	198 (3.7 %)
UCVA	logMAR	−0.023 (0.114)
BCVA	logMAR	−0.040 (0.076)
Objective SE	D	0.061 (0.480)
Subjective SE	D	−0.006 (0.075)
AL	mm	23.29 (0.76)
K value strong	D	43.9 (1.4)
K value weak	D	43.0 (1.3)
High C4	μm	0.118 (0.056)
Comma-like (Z3) C4	μm	0.104 (0.054)
Spherical-like (Z4) C4	μm	0.052 (0.029)
Spherical C4	μm	0.033 (0.024)
High C6	μm	0.359 (0.133)
Coma-like (Z3) C6	μm	0.285 (0.122)
Spherical-like (Z4) C6	μm	0.204 (0.093)
Spherical C6	μm	0.169 (0.092)
High O4	μm	0.105 (0.047)
Coma-like (Z3) O4	μm	0.091 (0.046)
Spherical-like (Z4) O4	μm	0.048 (0.024)
Spherical O4	μm	0.026 (0.027)
High O6	μm	0.377 (0.198)
Coma-like (Z3) O6	μm	0.297 (0.155)
Spherical-like (Z4) O6	μm	0.130 (0.161)
Spherical O6	μm	0.214 (0.152)

The data of both eyes were averaged for descriptive purpose

*BCVA* best-corrected visual acuity, *D* diopters, *High* total higher-order aberrations (HOAs), *K value* corneal refractive power, *UCVA* uncorrected visual acuity, *y.o.* years old, *Z* Zernike order, *C4* 4-mm pupil diameter corneal HOAs, *C6* 6-mm pupil diameter ocular HOAs, *O6* 6-mm pupil diameter ocular HOAs

*Frequency (percentage) for categorical variables

In the multivariable linear mixed model analysis with the axial length elongation of emmetropic children after one year as the dependent variable, the results showed that out of the 17 variables examined, 7 fixed effects were found to be significant (Table 4). These results demonstrated that school children of a certain sex (female), lower grade (younger), higher myopia, longer AL, smaller corneal coma-like and ocular spherical aberrations (SAs) (6-mm pupil), and greater ocular SAs (4-mm pupil) are at a higher risk of future AL elongation in emmetropic children.

**Table 4:** Linear mixed effects model for AL changes in emmetropic children after 1 year

	Estimated value	95% CI	*P* value
Grade	−0.0273	−0.0339, −0.0208	< 0.001
Sex			
Male	ref		
Female	0.0221	0.0049, 0.0392	0.01
Objective SE, D	−0.0135	−0.0195, −0.0076	< 0.001
AL, mm	0.0202	0.0063, 0.0340	0.004
Average K value, D	0.0072	−0.0029, 0.0174	0.16
Coma-like (Z3) 4C, μm	0.0019	−0.0030, 0.0068	0.44
Spherical-like (Z4) 4C, μm	−0.0004	−0.0049, 0.0040	0.85
Spherical 4C, μm	−0.0015	−0.0056, 0.0027	0.48
Coma-like (Z3+Z5) 6C, μm	−0.0049	−0.0096, −0.0001	0.04
Spherical-like (Z4+Z6) 6C, μm	0.0015	−0.0032, 0.0062	0.54
Spherical 6C, μm	0.0009	−0.0036, 0.0055	0.69
Coma-like (Z3) 4O, μm	0.0017	−0.0035, 0.0069	0.53
Spherical-like (Z4) 4O, μm	0.0037	−0.0011, 0.0084	0.13
Spherical 4O, μm	0.0063	0.0009, 0.0117	0.02
Coma-like (Z3+Z5) 6O, μm	−0.0020	−0.0069, 0.0029	0.42
Spherical-like (Z4+Z6) 6O, μm	0.0044	−0.0022, 0.0109	0.19
Spherical 6O, μm	−0.0092	−0.0162, −0.0023	0.009

Multivariate (N=5384)

*AL* axial length, *Average K* Average corneal refractive power, *CI* confidence interval, *D* diopters, *SE* spherical equivalent, *Z* Zernike order, *O4* 4-mm pupil diameter ocular higher-order aberrations (HOAs), *O4* 4-mm pupil diameter ocular higher-order aberrations (HOAs)

## Discussion

In the preliminary study, longer AL showed correlation with myopia progression. There was a correlation between AL elongation and the development of myopia. The findings in the primary study showed a significant relationship between AL elongation and sex, school grade, baseline AL, refraction, and several HOAs in emmetropic school children. Since the progression of myopia can be predicted by AL, the risk factors of AL elongation in emmetropic students may potentially serve as risk factors for the onset of myopia.

In the preliminary study, we found a strong correlation between the changes in AL elongation and in myopic refraction. Consistent with previous findings [25–29], it was demonstrated that longer AL in lower grades of school children is a risk factor for subsequent myopia progression and AL elongation. These findings suggest that the myopia progresses in proportion to the elongation of the AL.

There were no significant changes in corneal refractive power and ocular HOA from Grade 3 to Grade 6. As the eyeball enlarges with growth, the cornea flattens, leading to a reduction in HOAs [30]. It is possible that three years was too short a period to capture the changes in corneal flattening in the preliminary study. On the other hand, in school-age children, corneal shape changes only slightly, and myopia progresses as the AL increases and the focal point moves in front of the retina. This may explain why school-age myopia is axial myopia [31].

Sex, age, AL, and refraction were found to influence AL elongation in emmetropic children in the primary study. Consistent with previous meta-analyses [32, 33], girls and younger age are risk factors of myopia progression. The mechanism by which the sex of the subject affects myopia progression is not known. However, there are reports that women are at risk for myopia progression. Since girls stop growing earlier than boys, it is assumed that the orbital growth also stops earlier. This causes the eye to stop expanding evenly and quickly in girls, and the eye axis to elongate backward, which can lead to the progression of myopia. There are numerous reports that longer AL and myopic refraction are risk factors of AL elongation from many countries [26–29, 34, 35]. It would be even more useful if future myopia onset could be predicted directly from AL values.

In addition, several HOAs associate the AL elongation in emmetropic children in the primary study. As with previous studies [36–39], the present study shows that higher corneal coma-like aberrations inhibit AL elongation. Furthermore, SAs are also indicated to be related to myopia progression in this study. Higher ocular SAs of a 6-mm pupil may inhibit myopia progression, similar to coma-like aberrations, due to an increase in multifocality and apparent accommodations. In a previous study by Padmaja et al., the authors report on a novel contact lens that reduces relative peripheral retinal hyperopia, reducing myopia progression [40]. It is possible that more aberration at the peripheral retina may indicate a more inhibitory effect on myopia onset. Rum et al. designed a Defocus Incorporated Multiple Segments (DIMS) spectacle lens that imposes defocus on both the central and peripheral retina [41], and they report that the DIMS lens inhibited myopia progression in school children. We hypothesize that the increase in higher ocular SAs of a 6-mm pupil may suppress AL elongation by inducing a myopic blurred image at the peripheral retina.

Interestingly, opposite results were obtained in 6-mm and 4-mm diameter pupils. The higher ocular SAs of a 4-mm pupil present the risk of AL elongation. A pupil diameter of 4 mm is considered to reflect a more normal state of vision than a pupil diameter of 6 mm. It is suggested that blurred images in the central retina may affect myopia progression, and we believe that the higher ocular SAs of a 4-mm pupil causes a decrease in image quality at the central retina, thus promoting axial elongation (i.e., a form of deprivation myopia) [42, 43].

It should be noted that this study did have limitations. First, this study was conducted in the limited geographical area of Kyoto Prefecture, and was limited to Japanese children. Hence, the findings in this study pertain to only a specific region and are obviously not universally applicable, so further study over a wider area and a larger number of subjects is needed. Second, although this study defined myopia onset by ocular axial elongation, we aim to focus future studies on examining the significant differences with the SE changes.

To conclude, in school children, those with longer AL tend to experience more progression of myopia. Myopic refractive errors increase in proportion to AL elongation. Emmetropic children with an elongated AL are more likely to develop myopia. This study suggests associations of myopia onset with not only refraction, AL, age, and sex, but also corneal coma-like aberrations and ocular SAs. To prevent myopia onset, children with these risk factors require proactive guidance even if they are emmetropic.

## Supplementary Information

Below is the link to the electronic supplementary material.

Supplementary file1 (DOCX 58 KB)
