# Supplementary material for: Biometric risk factors for myopia onset in emmetropic school-age children
Source: Jpn J Ophthalmol. 2025 Jun 3;69(5):687–93. doi: 10.1007/s10384-025-01222-2 (PMC12390880; doi:10.1007/s10384-025-01222-2)
Supplement: Supplementary file 1 — Supplementary file1 (DOCX 58 KB) [file 10384_2025_1222_MOESM1_ESM.docx]

Supplemental Figure 1

*

*

*


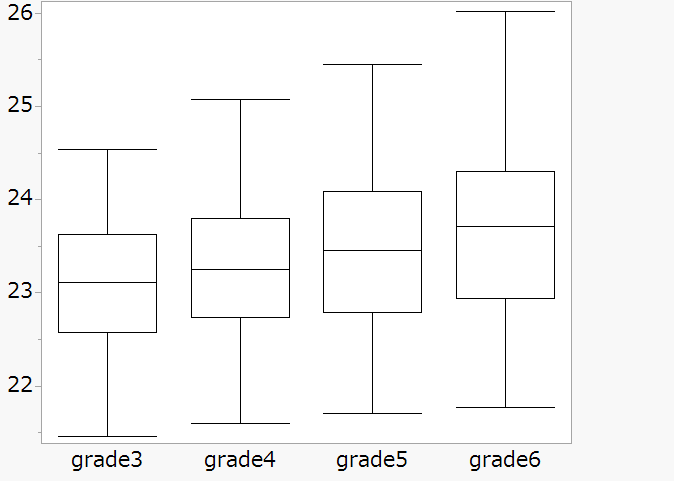


Axial Length (mm)

Supplemental Figure 2


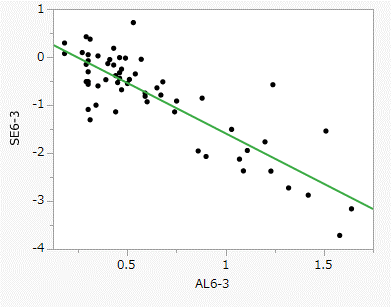


Axial Length changes (mm)

Refractive change (Diopter)

iopter)

Supplemental Figure legend

**(mm)**

Figure 1

Boxplot of axial length.

This is the trend of AL over each year, from grade3 to grade6, for the same cases. AL is significantly increasing every year. (*; p<0.05) The upper limit of the boxplot is the third quartile + 1.5×interquartile range (IQR), and the lower limit is first quartile - 1.5×IQR.

Figure 2

Correlation between axial length change and refraction change.

Correlation between AL change and refraction change over 3 years from grade3 to grade6 for the same cases.

Refractive changes = 0.5103002 - 2.0999116 x AL changes, R^2^=0.710124
